# Supplementary material for: Patterns of Intron Gain and Loss in Fungi
Source: PLoS Biol. 2004 Nov 30;2(12):e422. doi: 10.1371/journal.pbio.0020422 (PMC532390; doi:10.1371/journal.pbio.0020422)
Supplement: Table S1 — Also available at http://genes.mit.edu/NielsenEtAl/. (4.3 MB ZIP). [file pbio.0020422.st001.zip › NielsenEtAl/html/12.html]

AN1376.1.NCU04699.1.MG01295.1.FG08613.1


```
 CLUSTAL W (1.82) Multiple Sequence Alignments - Introns Inserted


Sequence 1: NCU04699.1	214 aa
Sequence 2: FG08613.1	223 aa
Sequence 3: MG01295.1	207 aa
Sequence 4: AN1376.1	180 aa
Alignment Length: 224 aa
Number Identitical Residues: 79 aa
Alignment Score (without introns) 4051


MG01295.1 	---MAPAPTPLGQLVDFSQESFVA1SSRDR0RPQPP---~-GCRTE~YHNKVLTRLFGGR
NCU04699.1	MPLVALGVADLFNFVDYSKTSLAI1SAAAI~AFNPTFWN2IVARRE1YRTKFLTRAFGGN
FG08613.1 	MGIMALGPQDFLDYVNYDKQSLYI1SAASI~AFNPLFWN2IVARQE1YHNKLLTKLFGGN
AN1376.1  	---MLRCP---LHLIRYSG-----~-----~--------~-----K1YRSHFLTRIFG-S
          	   :        . : :.                           : *:.:.**: **  

MG01295.1 	SQTACYFLAATIFSLGIVRDLLYERALRDQPAHPLLDNDLATYIGYALIGCGNILVVSST
NCU04699.1	AQVACYFLAVTIFGLGLVRDFLYERALRDQPSHPLLEGTYVKYAAYALLALGNLLVITST
FG08613.1 	SQVACYALAFTIFSLGMVRDLVFKNAISEQPTHPALVNDYMRAAAATLFAVGNILVVSST
AN1376.1  	PYRGCYFLAITIFSLGILRDHIYQQALEDQPYYAPVH---QPVLGGALFAVGSVLVLSSM
          	.  .** ** ***.**::** :::.*: :** :. :        . :*:. *.:**::* 

MG01295.1 	WQLGITGTFLGDYFGILMDHMVTGFPFNVTDAPMYYGSTMSFLGGALIYGKPAGVWLTAW
NCU04699.1	MRLGITGTFLGDYFGILMDGIVTGFPFNVTSAPMYYGSTMSFLGTALLYGKPAGLLLTAW
FG08613.1 	WRLGITGTFLGDYFGFLMDEMVTGFPFNVTGSPMYTGSTCSFLATSLWYGKPAGVLLTIW
AN1376.1  	YALGVTGTYLGDYFGILMDAPVTGFPFNVTGSPMYWGSTLNFLGVALYKGKVAGILLTAL
          	  **:***:******:***  *********.:*** *** .**. :*  ** **: **  

MG01295.1 	VLMVYKVALSYEN~PFTSEIYAKRERERAA-~---------EKTELKKTR
NCU04699.1	VLFVYIIAIQFEN~PFTAEIYAKRDRERAK-~---------AAGTSKKEL
FG08613.1 	VDIVYKLALRYED~PFTSEIYAKRERERAAG1WHGKATPFNRPGASQQA-
AN1376.1  	VFVLYWFALKWED2PFTAEIYAKRERERAK-~---------SKRGGKNQ-
          	* .:* .*: :*: ***:******:****                 ::
```
